# Therapy-resistant lymphomas may want nucleotides, but they just need to grow

**DOI:** 10.1172/JCI208384

**Published:** 2026-07-15

**Authors:** Carlos Carmona-Fontaine

**Affiliations:** Center for Genomics & Systems Biology, Department of Biology, New York University, New York, New York, USA.

## Abstract

Malignant cells must rapidly synthesize nucleotides to grow and proliferate. Antimetabolite chemotherapies throw a wrench in this process by administering decoy molecules resembling nucleotide precursors that cells cannot use, such as 6-mercaptopurine (6MP) and methotrexate. While this approach remains an essential tool in the treatment of lymphoblastic leukemias and B cell non-Hodgkin lymphomas, approximately 1 in 3 patients will eventually develop therapy-resistant malignancies. In this issue of the *JCI*, Yang et al. investigated the metabolic adaptations that enable therapy-resistant tumors to grow in the presence of these drugs. Using their previously described mouse model of MYC-driven large B cell lymphoma, they identified that increased expression of the vesicular oligopeptide and histidine transporter SLC15A3 drives dipeptide accumulation in therapy-resistant cells. In lieu of finding other ways to make more nucleotides, these adaptations force cell growth by boosting mTOR signaling. This cunning adaptation, however, is also a vulnerability that can be targeted clinically.

## Nucleotide deficiency–resistant lymphomas accumulate dipeptides by upregulating SLC15A3

When Jagger and Richards wrote the lines “You can’t always get what you want, but if you try sometime…you get what you need” in the Rolling Stones’ hit song “You Can’t Always Get What You Want (Satisfaction),” surely they were not thinking about cancer biology. But according to an article by Yang et al. in this issue of the *JCI*, these song lyrics neatly describe how malignant lymphomas become resistant to antimetabolite therapy (1). Antimetabolite therapies, including 6-mercaptopurine (6MP) and methotrexate, are both a long-standing treatment for malignant lymphomas and a possible therapeutic approach for cancers with limited treatment options, such as malignant rhabdoid tumors (2). Unfortunately, many lymphomas become resistant to these drugs (3), and the molecular adaptations enabling this resistance were not well understood.

MYC amplification is a prominent oncogenic driver in B cell malignancies (4). The Ruggero lab had previously developed a MYC-driven large B cell lymphoma mouse model where the deletion of phosphoribosyl pyrophosphate synthetase 2 (PRPS2), a rate-limiting enzyme in nucleic acid biosynthesis, mimics the effects of antimetabolite therapies (5). As expected, the absence of PRPS2 drastically reduces tumorigenesis, but about 40% of these mice eventually develop tumors, making this an elegant genetic model to study resistance to nucleotide deficiency.

In the present work, Yang et al. extended prior findings in the MYC-driven large B cell lymphoma model. They isolated MYC-driven lymphoma cells with or without the loss of *Prps2* and compared their metabolic steady states using untargeted metabolomics. They observed that *Prps2*-null lymphomas significantly accumulated dipeptides, most of which contained a glutamine or glycerol residue paired with an essential amino acid. Interestingly, the levels of these dipeptides were higher than in lymphomas with functional PRPS2 but were comparable to premalignant or wild-type B cells. In future work, it may be interesting to investigate the function of elevated peptides in normal B cells.

**Figure 1:**
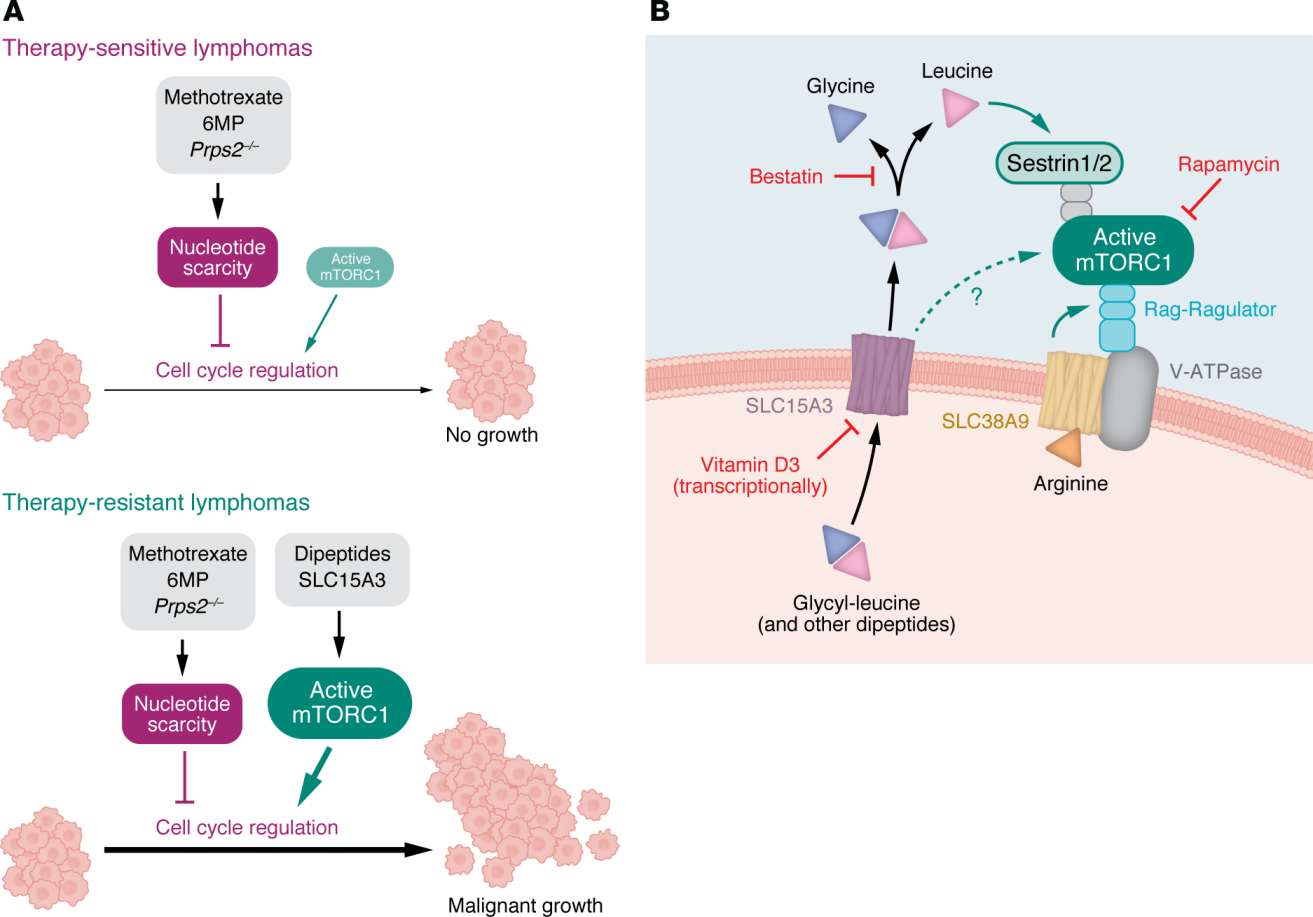
Antimetabolite-resistant lymphomas circumvent the need for nucleotides by activating mTOR. (**A**) Antimetabolite therapies like methotrexate and 6MP inhibit cell proliferation by reducing nucleotide biosynthesis, but many malignant lymphomas become resistant to these therapies. Yang et al. (1) used a *Prps2*^–/–^ model of MYC-driven large B cell lymphoma to model resistance to nucleotide deficiency. They found that therapy-resistant lymphomas did not develop an ability to fix nucleotide scarcity. Instead, they overrode the growth inhibition by antimetabolite therapies through mTOR activation, forcing cells to proliferate. mTORC1, mTOR complex 1. (**B**) Mechanistically, they showed that increased expression of SLC15A3 led to accumulation of dipeptides, including glycyl-leucine, in therapy-resistant lymphomas, driving mTORC1 activation. SLC15A3 may directly or indirectly activate this complex, and Yang et al.’s metabolomic analyses suggested that vitamin D3 may negatively regulate *Slc15a3* expression. Potential targets for therapeutic interventions are shown in red.

In parallel to their metabolomic comparisons, Yang et al. looked at transcriptional and translational changes and singled out the significant increase in *Slc15a3* mRNA levels in MYC-driven *Prps2*-null lymphomas. SLC15A3 is a proton-coupled oligopeptide and histidine transporter localized in endosomal and lysosomal vesicles. The authors then linked observations of dipeptide accumulation with enhanced *Slc15a3* expression, showing that SLC15A3 was responsible for the increase in dipeptide levels observed in therapy-resistant cells. In turn, the upregulation of SLC15A3 was essential for resistance to nucleotide deficiency in their MYC-driven lymphoma mouse model as well as essential for the emergence of 6MP resistance in human B cell lymphoma cells in culture. Providing clinical support to their model, biopsies from patients obtained before and after antimetabolite therapy showed a significant increase in SLC15A3 levels after treatment. One of the aspects that makes this work interesting is that the increases in SLC15A3 and dipeptides did not resolve the strained nucleotide biosynthesis induced by antimetabolite therapy. Instead, these increases counterbalanced the antiproliferative effects of nucleotide scarcity, with growth stimulation via the activation of mTOR signaling (Figure 1A).

## All you need is growth

As a vesicular nutrient transporter, SLC15A3 likely exchanges substrates between the cytosol and the lysosome. In unicellular eukaryotes, lysosomal transporters play a critical role as these organisms obtain a substantial portion of nutrients via endocytosis and use vacuoles as important nutrient storage (6). This central metabolic function of the lysosome is conserved in animal cells, including the prominent presence of vesicular nutrient transporters.

The central evolutionary role of lysosomes also extends to the activation of mTOR complexes such as mTORC1. This complex is physically tethered to lysosomes through interactions with cytosolic domains of lysosomal transmembrane proteins, such as vacuolar H^+^-ATPase (V-ATPase) (7). The lysosomal membrane provides a privileged location to sense nutrients engulfed by vesicles and acts as a landing pad where mTORC1 components and adaptors can cluster together. This physical proximity allows the integration of signals from nutrient sensors such as SLC38A9, which measures lysosomal levels of arginine (8, 9), or Sestrin2, which senses cytosolic levels of amino acids, such as leucine (10) (Figure 1B), with growth factors and other cellular inputs. The physical proximity of all these proteins also allows mTOR complexes to efficiently phosphorylate a range of downstream effectors (11, 12).

Yang and colleagues propose that SLC15A3 has a similar mTOR activation role in antimetabolite-resistant lymphoma cells. Through a series of clever experiments, they determined that these cells drastically increased mTORC1 signaling. Importantly, their data show that elevated dipeptides enhanced mTORC1 activation in resistant lymphomas and that reduction of SLC15A3 attenuated mTORC1 activity (1).

Together, their results support a model where oligopeptides transported by SLC15A3 activate mTORC1 to restore cell growth. Using a proximity ligation assay, the authors showed that in 6MP-resistant cells, SLC15A3 and mTORC1 are positioned within a few nanometers of each other, suggesting that SLC15A3 may directly activate mTORC1, as has been demonstrated for other lysosomal transporters, such as SLC38A9 (8, 9).

## Identifying therapeutic opportunities for patients with chemoresistant tumors

It is also possible that SLC15A3 indirectly activates mTORC1. For example, dipeptides transported into the cytosol via SLC15A3 may be cleaved by cytosolic peptidases, producing free amino acids, such as leucine — a well-known mTORC1 activator (10) and an abundant residue within the dipeptides reported in this study. If so, peptidase inhibitors such as bestatin or tosedostat may provide additional options to treat antimetabolite-resistant lymphomas. Along these lines, our recent report showed that solid tumors feed on oligopeptides by secreting an exopeptidase called CNDP2 (13). Secreted CNDP2 then hydrolyzes extracellular di- and oligopeptides, producing free amino acids that cells then take up. While there are obvious differences between the process described by Yang et al. and our findings, both studies uncover important functions for oligopeptides and open therapeutic avenues to target tumor growth and therapy resistance.

The activation of mTORC1 presents additional therapeutic opportunities for antimetabolite-resistant tumors. The mTOR inhibitor rapamycin and related drugs may prevent growth in therapy-resistant tumors with elevated SLC15A3. To test the effects of mTOR inhibition in B cell lymphomas, Yang et al. chose to treat a range of mouse B cell lymphoma models with RapaLink-1, a third-generation, bivalent mTOR inhibitor that strongly inhibits mTORC1 and mTORC2 (14). Consistent with their cell culture evidence, RapaLink-1 significantly and specifically increased survival in therapy-resistant lymphomas. These results provide an exciting path for a direct clinical application where patients who develop therapy-resistant tumors may be stratified according to SLC15A3 levels. Patients with clinically significant upregulation of SLC15A3 levels may benefit from treatment with mTOR inhibitors — many of which are approved by the US Food and Drug Administration. Since their data pointed to a specific role of mTORC1, it would be interesting to see if the effects they observed in their preclinical models would remain true when using inhibitors specific to this complex.

A third therapeutic angle is targeting the regulation of *Slc15a3* expression in antimetabolite-resistant tumors. By analyzing the promoter of this gene, the authors predicted that the nuclear vitamin D receptor regulates the *Slc15a3* locus (1). Interestingly, their metabolomics data revealed that active vitamin D3 — a strong vitamin D receptor ligand — was significantly reduced in therapy-resistant cells, suggesting that vitamin D3 may act as a negative regulator of *Slc15a3* expression. Consistent with their analyses, antimetabolite-resistant lymphoma cells were sensitive to vitamin D3 application, which significantly reduced SLC15A3 levels, activated mTORC1, and increased cell death.

## But what about the nucleotides that cells want?

It is reasonable to predict that cells may acquire antimetabolite therapy resistance by increasing or modifying nucleotide biosynthesis pathways to become insensitive to antimetabolites. Instead Yang et al. showed that these malignant cells do not obtain the additional nucleotides that they “want,” but they do get what they “need”: growth-promoting signals. Thus, antimetabolite therapies trigger stress responses that halt cell growth much earlier than when nucleotides become *stoichiometrically* limited for growth. This observation is important because if nucleotides were truly limiting for growth, the activation of growth-stimulating pathways such as mTORC1 would not result in effective cell proliferation.

As lymphoma cells balance positive and negative growth signals, antimetabolite therapies induce nucleotide scarcity stress signals leading to growth arrest. This work shows that these cells can gain therapy resistance simply by overriding antiproliferative signals using the sheer power of mTOR activation (Figure 1A). The work by Yang et al. not only provides us a careful molecular dissection of how MYC-driven lymphomas become resistant to antimetabolite therapies, but it also reveals key vulnerabilities that can be readily targeted in the clinic using existing and approved drugs.

## Conflict of interest

The author has declared that no conflict of interest exists.

## Funding support

- Innovator Award from the Pew Biomedical Foundation, 39047.

## Version 1 07/15/2026

Electronic publication

## References

[1] Yang et al (2026). SLC15A3-mediated dipeptide metabolism confers antimetabolite resistance in lymphoma via mTORC1 activation. J Clin Invest.

[2] Kes MMG (2025). Metabolic profiling of patient-derived organoids reveals nucleotide synthesis as a metabolic vulnerability in malignant rhabdoid tumors. Cell Rep Med.

[3] Wilson WH (2006). Drug resistance in diffuse large B-cell lymphoma. Semin Hematol.

[4] Ott G (2013). Understanding MYC-driven aggressive B-cell lymphomas: pathogenesis and classification. Blood.

[5] Cunningham JT (2014). Protein and nucleotide biosynthesis are coupled by a single rate-limiting enzyme, PRPS2, to drive cancer. Cell.

[6] Wideman JG (2014). The cell biology of the endocytic system from an evolutionary perspective. Cold Spring Harb Perspect Biol.

[7] Zoncu R (2011). mTORC1 senses lysosomal amino acids through an inside-out mechanism that requires the vacuolar H(+)-ATPase. Science.

[8] Rebsamen M (2015). SLC38A9 is a component of the lysosomal amino acid sensing machinery that controls mTORC1. Nature.

[9] Wang S (2015). Metabolism. Lysosomal amino acid transporter SLC38A9 signals arginine sufficiency to mTORC1. Science.

[10] Wolfson RL (2016). Sestrin2 is a leucine sensor for the mTORC1 pathway. Science.

[11] Battaglioni S (2022). mTOR substrate phosphorylation in growth control. Cell.

[12] Panwar V (2023). Multifaceted role of mTOR (mammalian target of rapamycin) signaling pathway in human health and disease. Signal Transduct Target Ther.

[13] Guzelsoy G (2025). Cooperative nutrient scavenging is an evolutionary advantage in cancer. Nature.

[14] Rodrik-Outmezguine VS (2016). Overcoming mTOR resistance mutations with a new-generation mTOR inhibitor. Nature.

